# Description and Validation of TAVIApp: A Novel Mobile Application for Support of Physicians in the Management of Aortic Stenosis—Management of Aortic Stenosis with TAVIApp

**DOI:** 10.1155/2017/9027597

**Published:** 2017-11-15

**Authors:** Ciro Indolfi, Jolanda Sabatino, Salvatore De Rosa, Annalisa Mongiardo, Pietrantonio Ricci, Carmen Spaccarotella

**Affiliations:** ^1^Division of Cardiology, Department of Medical and Surgical Sciences, Magna Græcia University, Catanzaro, Italy; ^2^URT-CNR, Department of Medicine, Consiglio Nazionale delle Ricerche, Catanzaro, Italy; ^3^Institute of Forensic Medicine, Department of Medical and Surgical Sciences, Magna Græcia University, Catanzaro, Italy; ^4^The Ethics Committee, Regione Calabria, Sezione Area Centro, Catanzaro, Italy

## Abstract

**Background:**

Aortic stenosis (AS) is the most common heart valve disease in developed countries. The advent of transcatheter aortic valve implantation (TAVI) significantly improved patients' outcome but made clinical management more complex. The aim of the present study was to describe TAVIApp, a mobile app we developed to guide the management of AS, and test its efficacy.

**Methods and Results:**

Clinical cases comprising 42 patients with AS were blindly evaluated by (A) an interventional cardiologist, assisted by the Heart Team (EXPERT), (B) young residents in cardiology, and (C) a young resident supported by TAVIApp. There was poor concordance between Group A and Group B with low performance by young residents (*k* = 0.52; *p* < 0.001). However, concordance increased to an optimal value when young residents were supported by TAVIApp (*k* = 1.0; *p* < 0.001) for the diagnosis of severe AS and eligibility assessment. Furthermore, regarding the selection of the most appropriate prosthesis size, concordance to Group A was poor without TAVIApp support (Group B) (*k* = 0.78; *p* = 0.430), but excellent with TAVIApp (*k* = 1.0; *p* < 0.001).

**Conclusions:**

This study is the first describing and validating a new mobile application to support the management of AS. TAVIApp supports cardiologists in the evaluation of stenosis severity, eligibility for TAVI or AVR, and selection of the most appropriate prosthesis size in individual patients.

## 1. Introduction

Aortic valve stenosis (AS) is currently the most common heart valve disease in developed countries [1]. The most frequent etiology is the progressive degeneration of valve cusps, with frequently associated calcifications [2]. Consequently, the increase in the average lifespan in the general population largely contributes to its growing prevalence (2–7% in subjects over 65 years of age) [3].

Surgical aortic valve replacement (AVR) was traditionally the only treatment option, until transcatheter aortic valve implantation (TAVI) was introduced as a valuable alternative to surgical AVR. Initially reserved to nonoperable patients, indications for TAVI have been progressively enlarged to include high surgical risk patients and, more recently, those at intermediate surgical risk [4].

The large diffusion of smartphones and tablets in the last years has propelled the development of a new generation of software for mobile applications (apps). Use of mobile apps has gained an incredible momentum within the last few years, reaching the healthcare field. “Healthcare” apps are currently being used in several ways, both by patients and by physicians. Tens of thousands of medical apps are currently available on the most popular mobile application stores [5], and the widespread adoption and use of mobile technologies are opening new and innovative ways to improve health and healthcare delivery [6–19]. In particular, smartphone apps have been developed to guide the diagnosis and treatment of arterial hypertension, atrial fibrillation, or supraventricular tachycardia, to support interventions for smoking cessation, weight loss programs, and exercise schemes in cardiac rehabilitation [20–28]. Given the large amount of information that should be taken into account when evaluating patients with AS, smartphone-based software would be helpful. In this context, although some mobile apps are available on the mobile marketplace, they are either limited to educational purposes, specifically focused on risk stratification, or dedicated to list the transcatheter bioprostheses to be matched to the dysfunction surgical bioprostheses of specific patients (prostheses sizing for the valve-in-valve procedures). Furthermore, none of these apps has undergone a scientific evaluation process. On the contrary, TAVIApp is the only mobile app available to date that provides a decision support system for the management of patients with AS, encompassing a support both to establish the diagnosis of severe AS and to select the correct prosthesis size for TAVI procedures.

Hence, the aim of the present study was to describe TAVIApp, a mobile application we designed to support physicians during the diagnostic process of AS and for the selection of the most appropriate self-expanding aortic prosthesis, to test the feasibility and efficacy of its clinical use.

## 2. Methods

The diagnostic algorithm implemented in the app was based on the latest guidelines of the European Society of Cardiology and of the American College of Cardiology/American Heart Association on AS [29, 30].

Besides guiding the diagnostic process, the application assesses the clinical and anatomical suitability for TAVI and supports physicians in the selection of the appropriate CoreValve size [31, 32], based on data obtained from echocardiography and multidetector computed tomography (MDCT), according to manufacturer indications (Medtronic Inc., Minneapolis, MN, USA).

### 2.1. Development of TAVIApp

**Figure 1:**
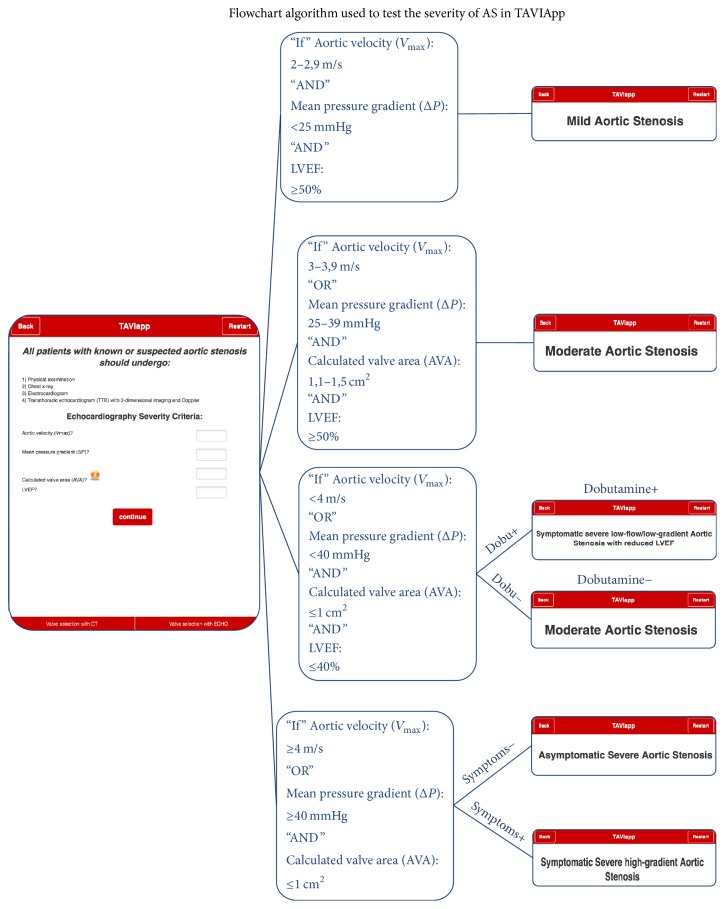
Algorithm flowchart for the diagnostic assessment of aortic stenosis severity. The figure describes the algorithm underlying the decision support process with the application for the diagnosis of aortic stenosis and assessment of disease severity.

The Cardiology Division and the Engineering Department of the Magna Græcia University developed the mobile application called TAVIApp, which is currently available for free download on the Apple App Store and on Google Play Store. The algorithm flowchart used to test the severity of AS in TAVIApp is reported in Figure 1.

TAVIApp has been developed by using Apache Cordova API (Application Programming Interface). Apache Cordova is a set of device APIs that allow a mobile device app developer to access native device functions with the general structure of a web app. When using the Cordova APIs, an app can be built without any native code (Java, Objective-C, etc.) from the app developer. Apache Cordova is a set of device APIs developed within the Apache Software Foundation (ASF). The main reasons why Cordova APIs were used to develop TAVIApp were (a) the need to model the entire decision-making process provided by the medical team in a single programming language; (b) the need to avoid rewriting the application code in different languages to make it compatible with different operating systems (e.g., iOS, Android); (c) the need to keep system maintenance as easy as possible, especially during the testing and debugging of the application itself.

In particular, we used the “PhoneGap” framework that allows creating mobile apps using standardized web APIs for any platform. By using PhoneGap application, we exploited all the power and simplicity of expression languages like HTML, CSS, and JavaScript and a set of APIs provided by the framework to access the native functionality of the device, as already previously described [33].

### 2.2. Evaluation of TAVIApp

The usefulness and efficacy of the mobile application were validated in patients under evaluation for TAVI at the Division of Cardiology, Magna Græcia University, Catanzaro, Italy. In particular, forty-two consecutive patients were blindly evaluated by means of the following approaches: (A) an interventional cardiologist with long-standing experience in TAVI procedures with the Heart Team, also including a heart surgeon and an anesthesiologist (EXPERT), (B) two young residents in cardiology with no direct experience in TAVI, and (C) a young resident in cardiology with no direct experience in TAVI who evaluated all patients using TAVIApp. Physicians from all three groups had free access to all clinical data, including echocardiography, and MDCT in both cases and were asked to evaluate the severity of AS, to verify the indication for percutaneous treatment, and to select the most appropriate prosthesis size. Results obtained by the three groups were compared to each other to assess the efficacy of the mobile application.

### 2.3. Conventional Evaluation of Patients with AS at the Magna Græcia University (EXPERT)

The evaluation provided by the EXPERT Interventional Cardiologist was based on the following practice guidelines established at the Magna Græcia University: patients were considered as potential candidates for implantation of a CoreValve prosthesis (Medtronic CoreValve, Minneapolis, Minnesota, USA) if they had severe symptomatic AS and were considered at high or prohibitive surgical risk by the heart surgeon, as described elsewhere [3, 31, 32, 34–38].

The selection of the size of the valve prosthesis was based on the measurements of the aortic valve annulus obtained by CT, according to the manufacturer's sizing recommendations, to provide adequate anchoring and sealing between the prosthesis and the annulus.

**Figure 2:**
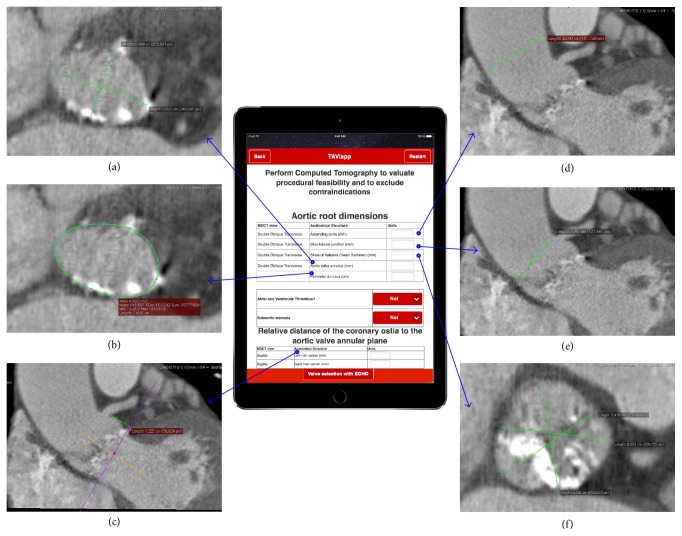
Selection of prosthesis size with TAVIApp using CT scan results. The figure reports a screenshot from TAVIApp (center) where the user can input the following results of CT scan measurements to be used for the selection of the most appropriate prosthesis size: (a) aortic valve annulus diameter; (b) aortic valve perimeter; (c) distance between the coronary ostia and the aortic valvular plane; (d) diameter of the ascending aorta; (e) aortic diameter at the sinotubular junction; (f) aortic diameter at the valsalva sinuses.

**Figure 3:**
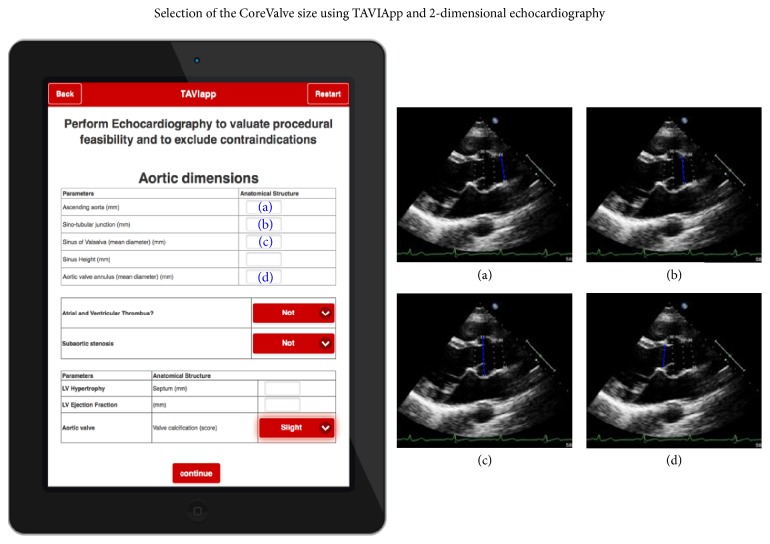
Selection of prosthesis size with TAVIApp using 2D echocardiography results. The figure reports a screenshot from TAVIApp where the user can input two-dimensional echocardiography measurements. When a CT scan is not available, TAVIApp selects the most appropriate CoreValve size using the following 2D echocardiography data: (a) diameter of the ascending aorta; (b) aortic diameter at the sinotubular junction; (c) aortic diameter at valsalva sinuses; (d) mean diameter of the aortic valve annulus.

In case of unavailability of CT imaging, the prosthesis can be sized based on 2D echocardiographic imaging [30]. For these reasons, TAVIApp is able to suggest the most appropriate prosthesis size on the basis of CT scan results (Figure 2) or, in lack thereof, with the measurements obtained with 2D echocardiography (Figure 3).

### 2.4. Main Outcome for Validity

The main outcome of the present evaluation was the classification match (CM) between the output obtained by study groups—Groups B and C—compared to Group A (EXPERT), for each of the following: (1) assessment of AS severity and (2) prosthesis size. Concordance rates between the study groups were tested using Cohen's kappa test, as described elsewhere [33]. Briefly, we tested the null hypothesis that evaluation of the 42 cases by the two groups under comparison yielded different results (i.e., not concordant).

Statistical analyses were performed using SPSS version 20.0 for Windows. All statistics were two-tailed and *p* values < 0.05 were considered statistically significant. The study protocol was approved by the local competent Ethics Review Board.

## 3. Results

### 3.1. Patients' Characteristics

Clinical features and imaging results of all patients included are shown in Table 1. The mean transvalvular pressure gradient was 44.04 ± 11.90 mmHg and the mean aortic valve area (AVA_Go_), as calculated by means of the Gorlin equation, was 0.71 ± 0.17 cm^2^. The AVA could be determined by means of the continuity equation (AVA_ce_) only in 36 cases (86%), due to suboptimal Doppler tracings in patients with severe aortic valve calcification or pulmonary emphysema (mean: 0.79 ± 0.19 cm^2^). At MDTC, the mean aortic valve annulus perimeter was 7.54 ± 1.05 cm, while the mean aortic valve annulus diameter was 23.23 ± 2.90 mm.

### 3.2. Evaluation of TAVIApp

To evaluate the function of the TAVIApp system, the Engineering Department of the UMG conducted a preliminary test set to check the stability of the software and the data collection server. Furthermore, the whole system was closely monitored during the entire study.

During the testing phase, we experienced two different classes of bugs: common errors occurring during the development phase, such as suboptimal graphical interface on the device screen, and misinterpretation of the clinical algorithm by the developers were promptly recognized and resolved before patients' testing.

For the first class of bugs, the developing team redesigned a specific element of the software to solve all issues during the test phase. For the second class, weekly debugging sessions were scheduled with both the medical developers and the engineering team to grab real-time feedback and speed up the debugging process.

**Figure 4:**
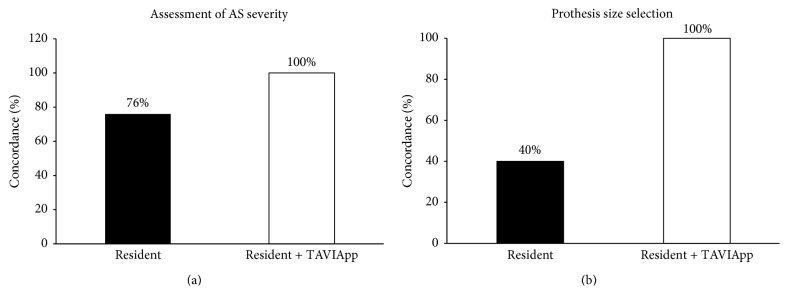
Classification match between the study groups. The bar graph shows the concordance rate for Group B (young residents) and Group C (young residents using TAVIApp) for the assessment of aortic stenosis severity (a) and selection of the most appropriate prosthesis size (b) as compared to Group A (EXPERT).

After this initial test phase, the efficacy of TAVIApp was finally evaluated against the EXPERT Interventional Cardiologist, as described above. We found that young residents that evaluated the clinical cases without using TAVIApp had a classification match of 76%, with a poor concordance index (*k* = 0.52; *p* < 0.001) compared to Group A (EXPERT), while there was no difference in the classification match for the assessment of severe AS between Group C (young residents using TAVIApp) and Group A (EXPERT), resulting in an optimal concordance index (*k* = 1.0; *p* < 0.001) (Figure 4(a)). Similarly, facing the task to select the most appropriate prosthesis size, young residents that evaluated the clinical cases without using TAVIApp (Group B) had a substantially lower classification match of 40%, with poor concordance (*k* = 0.078; *p* = 0.430) compared to Group A (EXPERT), while no difference was found between Group C (young residents using TAVIApp) and Group A (EXPERT), resulting in optimal concordance (*k* = 1.0; *p* < 0.001) (Figure 4(b)). In this latter comparison, all three groups could not identify a specific prosthesis size in 4 cases. In fact, the TAVIApp output indicated borderline measurements, and even the EXPERT could not select definitely a specific size. In both cases, the actual prosthesis size was selected after intraprocedural measurement by means of the valvuloplasty balloon, inflated at the aortic annulus during contrast medium injection. Finally, no difference was found between the groups in the choice of vascular access (*p* < 0.001).

## 4. Discussion

The main findings of the present study are as follows: (1) TAVIApp is a reliable support to help the diagnostic process of AS by an inexperienced cardiologist, also including identification of low-flow, low-gradient AS, and (2) TAVIApp is a useful decision support to select the appropriate prosthesis size for TAVI.

Currently, there are no support tools to help physicians in this complex evaluation in the busy clinical setting. TAVIApp aids clinicians with the above described dual assessment, incorporating evidence-based decision support for the diagnosis of severe aortic stenosis and technical selection criteria to select the most appropriate prosthesis size for which a specific patient qualifies.

This application was developed both for the Android and for Apple platforms. The development of a mobile app as a decision supporting system for AS could be particularly useful in a rapidly aging society, with a rapid increase in the number of patients with AS. Results of the present study support the use of TAVIApp as a decision support system for physicians managing patients with AS that are potential candidates of TAVI. Evaluation of the surgical risk in patients with AS is of key importance for the selection of the most appropriate treatment option. Although some risk scores are available, such as the logistic EuroScore and the Society of Thoracic Surgeons (STS) risk score [38], the use of these is not endorsed by current practice guidelines, which recommend instead that a multidisciplinary heart team should select the best procedural strategy for any single patient [29]. For this reason, the above referred risk scores were not included in TAVIApp.

The U.S. Food and Drug Administration (FDA) encourages the development of medical mobile apps that improve healthcare processes and provide patients and healthcare professionals with valuable health information. The FDA also has a public health responsibility to oversee the safety and effectiveness of medical devices including mobile medical apps [5–7]. Similarly, the European Commission recently published a green paper on mobile health to promote and regulate the use of mobile health technologies [39]. More recently, also the European Society of Cardiology (ESC) recognized the potential impact of mHealth in cardiovascular medicine [40]. For this reason, mobile apps to be used as a support in healthcare processes should be tested in real-life scenarios. In line with these recommendations, the validity of TAVIApp was tested on a set of patients' data sets. Results are promising based on our preliminary study, although further research is needed to confirm these findings.

### 4.1. Limitations of TAVIApp

A medical application cannot substitute clinical judgment. This is particularly true in specific conditions such as the case of erroneous measurements of CT perimeter or when the aortic dimensions are not indicative of a definite prosthesis size. In these cases, aortography with an inflated balloon can be helpful to select the right prosthesis size in a single patient. Of course, the impact of TAVIApp use could vary depending on the level of experience of the physicians and the clinical management processes used at single centers. Nevertheless, the aim of the present evaluation was limited to the demonstration, as a proof of concept, that the decision support provided by TAVIApp might be of help to the clinician.

## 5. Conclusions

In conclusion, the present study is the first one that describes and validates a new mobile application to help the decision-making process in patients with AS. In particular, TAVIApp helps the cardiologist in the diagnostic process to evaluate stenosis severity, including the case of low-flow and low-gradient AS, and supports the physician in the selection of the most appropriate prosthesis size.

## Figures and Tables

**Table 1:** Baseline echocardiographic, CT, and procedural variables.

	All studied patients (*n* = 42)
*Echocardiographic variables*	
Peak velocity (m/s)	4.08 ± 0.6
Mean aortic gradient (mmHg)	44.04 ± 11.9
AVA (cmq)	0.79 ± 0.19
LVEF (%)	50.28 ± 7.6
Septum (mm)	12.4 ± 1.1
*Computed tomography variables*	
Ascending aorta (mm)	33.04 ± 4.1
Sinotubular junction (mm)	26.45 ± 3.5
Sinus of valsalva (mean diameter) (mm)	31.10 ± 4.1
Aortic valve annulus (mean diameter) (mm)	23.23 ± 2.9
Perimeter (cm)	7.54 ± 1.05
Moderate valve calcification	16 (38)
Severe valve calcification	5 (12)
Distance of LM ostium to valve annular plane (mm)	13.62 ± 2.2
Distance of RM ostium to valve annular plane (mm)	15.00 ± 3.2
*Procedural variables*	
* Approach*	
Transfemoral	42 (100)
Transapical	None
* Bioprosthesis CoreValve diameter (mm)*	
23	2 (5)
26	14 (33)
29	20 (48)
31	6 (14)

Values are mean ± SD, or *n* (%). AVA: aortic valve area; LVEF: left ventricular ejection fraction; LM: left main; RCA: right coronary artery.

## Abbreviations

AS: Aortic valve stenosis
AVR: Surgical aortic valve replacement
TAVI: Transcatheter aortic valve implantation
MDCT: Multidetector computed tomography
API: Application Programming Interface
ASF: Apache Software Foundation
HTML: Hypertext markup language
CSS: Cascading style sheets
LVEF: Left ventricular ejection fraction
CM: Classification match
AVA: Aortic valve area
EuroScore: European System for Cardiac Operative Risk Evaluation
STS: Society of Thoracic Surgeons
FDA: U.S. Food and Drug Administration
MIUR: Ministero dell'Istruzione, dell'Università e della Ricerca (Italian Ministry of Education, Universities and Research).
